# Supplementary material for: Association of Acculturation Status with Longitudinal Changes in Health-Related Quality of Life—Results from a Cohort Study of Adults with Turkish Origin in Germany
Source: Int J Environ Res Public Health. 2021 Mar 10;18(6):2827. doi: 10.3390/ijerph18062827 (PMC7999343; doi:10.3390/ijerph18062827)
Supplement: Supplementary file 1 [file ijerph-18-02827-s001.zip › Supplementary_Files/Supplementary_Table_1.docx]

Supplementary Table S1. Participants’ characteristics by acculturation status.

|  | **Integration** (n=49) | **Assimilation** (n=84) | **Separation** (n=71) | **Marginalization** (n=35) |
| --- | --- | --- | --- | --- |
| **Age groups (%)** |  |  |  |  |
| 20-29 | 14.3 | 23.8 | 9.9 | 28.6 |
| 30-39 | 26.5 | 27.4 | 16.9 | 25.7 |
| 40-49 | 32.7 | 36.9 | 38.0 | 34.3 |
| 50-59 | 14.3 | 7.1 | 21.1 | 11.4 |
| 60-69 | 12.2 | 4.8 | 14.1 | 0.00 |
| **Gender (%)** |  |  |  |  |
| Male | 49.0 | 46.4 | 29.6 | 37.1 |
| Female | 51.0 | 53.6 | 70.4 | 62.9 |
| **Country of birth (%)** |  |  |  |  |
| Turkey | 75.5 | 58.3 | 83.1 | 77.1 |
| Germany | 20.4 | 40.5 | 9.9 | 20.0 |
| Missing | 4.1 | 1.2 | 7.0 | 2.9 |
| **Education (%)** |  |  |  |  |
| Low | 16.3 | 26.2 | 33.8 | 31.4 |
| Medium | 49.0 | 32.1 | 23.9 | 40.0 |
| High | 34.7 | 40.5 | 35.2 | 20.0 |
| Missing | 0.0 | 1.2 | 7.0 | 8.6 |
| **Income (%)** |  |  |  |  |
| <1000 Euro | 14.3 | 10.7 | 18.3 | 31.4 |
| 1000 - <2500 Euro | 42.9 | 41.7 | 47.9 | 37.1 |
| ≥2500 Euro | 26.5 | 39.3 | 19.7 | 17.1 |
| **PCS (mean, SD)** |  |  |  |  |
| Baseline | 45.7 (10.6) | 49.1 (9.7) | 44.2 (9.7) | 47.3 (8.0) |
| Follow-up | 44.8 (9.9) | 49.7 (9.8) | 42.9 (9.4) | 46.2 (9.9) |
| **MCS (mean, SD)** |  |  |  |  |
| Baseline | 45.1 (12.1) | 48.7 (9.7) | 42.9 (10.8) | 42.4 (13.1) |
| Follow-up | 42.7 (10.4) | 44.6 (11.1) | 40.8 (10.0) | 41.5 (10.8) |

Numbers are percentages or means (SD); SD: standard deviation; PCS: physical component summary score; MCS: mental component summary score;
