# Supplementary material for: Association of Acculturation Status with Longitudinal Changes in Health-Related Quality of Life—Results from a Cohort Study of Adults with Turkish Origin in Germany
Source: Int J Environ Res Public Health. 2021 Mar 10;18(6):2827. doi: 10.3390/ijerph18062827 (PMC7999343; doi:10.3390/ijerph18062827)
Supplement: Supplementary file 1 [file ijerph-18-02827-s001.zip › Supplementary_Files/Supplementary_Table_2.docx]

**Supplementary Table S2** Change over time in health related quality of life and acculturation status by sex (hierarchical linear regression).

|  | **Men** | | **Women** | |
| --- | --- | --- | --- | --- |
| **N** (observations) | 220 | | 385 | |
|  | **PCS** | **MCS** | **PCS** | **MCS** |
| **Change over time** | 0.4 | -3.5* | 0.6 | -4.9** |
|  | [-2.7,3.4] | [-6.8,-0.1] | [-2.7,3.8] | [-8.5,-1.3] |
|  |  |  |  |  |
| **Acculturation status** (Ref. Assimilation) | | | | |
| Integration | -2.6 | -1.3 | -1.8 | -5.6* |
|  | [-7.2,1.9] | [-6.5,3.9] | [-6.8,3.1] | [-11.0,-0.1] |
|  |  |  |  |  |
| Separation | 0.4 | -5.1 | -3.8 | -3.3 |
|  | [-4.5,5.3] | [-10.7,0.6] | [-7.8,0.3] | [-7.8,1.2] |
|  |  |  |  |  |
| Marginalisation | -2.1 | -7.2* | -0.1 | -1.7 |
|  | [-7.9,3.7] | [-13.8,-0.5] | [-5.3,5.1] | [-7.5,4.1] |
|  |  |  |  |  |
| **Time by acculturation** (Ref. Time#Assimilation) | | | | |
| Time#Integration | -0.9 | -1.0 | -1.9 | 5.1 |
|  | [-6.0,4.3] | [-6.6,4.6] | [-7.3,3.6] | [-1.1,11.3] |
|  |  |  |  |  |
| Time#Separation | 1.1 | 0.6 | -2.8 | 2.5 |
|  | [-4.3,6.5] | [-5.3,6.5] | [-7.3,1.6] | [-2.6,7.6] |
|  |  |  |  |  |
| Time#Marginalisation | 0.6 | 4.8 | -2.3 | 2.2 |
|  | [-5.8,6.9] | [-2.1,11.7] | [-8.0,3.5] | [-4.3,8.7] |
|  |  |  |  |  |

Adjusted for age, sex, education and income; 95% confidence intervals in brackets; *p<0.05, **p<0.01; PCS: physical component summary score; MCS: mental component summary score;
